# Fasting and time of day independently modulate circadian rhythm relevant gene expression in adipose and skin tissue

**DOI:** 10.1186/s12864-018-4997-y

**Published:** 2018-09-07

**Authors:** Alexessander Couto Alves, Craig A. Glastonbury, Julia S. El-Sayed Moustafa, Kerrin S. Small

**Affiliations:** Department of Twin Research and Genetic Epidemiology, King’s College London, St Thomas’ Campus, Westminster Bridge Road, London, SE1 7EH UK

**Keywords:** Gene expression, Fasting, Circadian rhythm, Gene x environment, eQTL, Adipose, Skin

## Abstract

**Background:**

Intermittent fasting and time-restricted diets are associated with lower risk biomarkers for cardio-metabolic disease. The shared mechanisms underpinning the similar physiological response to these events is not established, but circadian rhythm could be involved. Here we investigated the transcriptional response to fasting in a large cross-sectional study of adipose and skin tissue from healthy volunteers (*N* = 625) controlling for confounders of circadian rhythm: time of day and season.

**Results:**

We identified 367 genes in adipose and 79 in skin whose expression levels were associated (*FDR < 5%*) with hours of fasting conditionally independent of time of day and season, with 19 genes common to both tissues. Among these genes, we replicated 38 in human, 157 in non-human studies, and 178 are novel associations. Fasting-responsive genes were enriched for regulation of and response to circadian rhythm. We identified 99 genes in adipose and 54 genes in skin whose expression was associated to time of day; these genes were also enriched for circadian rhythm processes. In genes associated to both exposures the effect of time of day was stronger and in an opposite direction to that of hours fasted. We also investigated the relationship between fasting and genetic regulation of gene expression, including GxE eQTL analysis to identify personal responses to fasting.

**Conclusion:**

This study robustly implicates circadian rhythm genes in the response to hours fasting independently of time of day, seasonality, age and BMI. We identified tissue-shared and tissue-specific differences in the transcriptional response to fasting in a large sample of healthy volunteers.

**Electronic supplementary material:**

The online version of this article (10.1186/s12864-018-4997-y) contains supplementary material, which is available to authorized users.

## Background

Time restricted and Calorie restricted diets are orthogonal interventions that constraint when and what is eaten. Calorie restriction is among the most reproducible life-prolonging interventions across taxa [1]. Intermittent and periodic fasting are two modalities of diet restriction that improve many cardio-metabolic risk factors and may increase healthspan in humans [2]. Surprisingly, time restricted produces strikingly similar results to calorie-restricted diet [3], despite only restricting the period when meals are consumed and not calorie intake. The reasons behind this have just started to be elucidated, but may involve the circadian rhythm [4]. The circadian rhythm controls a variety of physiological processes, including metabolism, sleep-wake cycle, and feeding. Central to these rhythms is the circadian clock, a transcription-translation feedback loop that can sustain an approximately 24-h cycle. The central clock is located in the suprachiasmatic nuclei but other tissues also have clocks, e.g. heart, liver, kidney and adipose tissue [5]. The phase of the central clock is synchronized by light, but other factors such as mealtimes also entrain the phase of central and peripheral circadian clocks [6]. Both the absence and periodicity of mealtimes induced by intermittent fasting and time restricted diets may improve synchronization among central and peripheral clocks, which may lead to increased amplitude of clock gene expression [2]. Increased amplitude of the circadian rhythm oscillations is hypothesised to improve the overall health and cardio-metabolic status and disruption of circadian rhythm, e.g. shift work can lead to cardio metabolic conditions [2]. However, it is not clear how exactly this contributes to health status [7] or how to reconcile this with the pathogenesis of cardio metabolic disease, particularly the role of diet in connection with risk factors such as triglycerides, cholesterol, glucose, and insulin. Downstream pathways in peripheral tissues are plausible mediators of the circadian rhythm effect on health status, but the genes and the biological processes involved remain elusive.

One strategy to identify the molecular mechanisms mediating the effects of fasting on health is to identify fasting induced changes in gene expression across the body. Previous studies of the relationship of gene expression with fasting and calorie restriction investigated adipose, blood, skeletal muscle, liver, and mucosa gene expression by contrasting controls with fasting cases [8–10] or calorie restricted individuals [10–19], or by contrasting different times of the day [20]. However, none of these studies looked simultaneously at the effects of hours fasting and time of day on gene expression. Hence, it is not yet clear whether the effect of fasting is conditionally independent of the effect of time of day on circadian rhythms. Likewise, it is not yet clear if fasting and time of day response is independent of cardio metabolic risk factors. Other technical and design issues are also noteworthy, previous studies did not adjusted for the effects of seasonality, body mass index (BMI) and age, had smaller sample sizes (ranging from 4 to 311), used microarray or panels of genes, all of which may confound or bias the exploration of genes across the dynamic range of expression levels. Most importantly, skin transcriptome-wide gene expression response to hours fasting and time of day is yet uncharacterized.

Here we conducted an RNA-Seq gene expression association study on hours fasting adjusting for time of day, seasonality, BMI and age in adipose tissue and skin from 625 female twins from the TwinsUK registry. Adipose tissue plays a systemic role in metabolism, in the reproductive axis and in immune system homeostasis [21]. Skin is the largest organ and has an important systemic role on metabolism and the immune system [22] partly due to vitamin D synthesis. Circulating levels of vitamin D are associated with diabetes, cardiovascular disease, cancer, and osteoporosis [23]. Our aims are: 1) to map the pathways that independently respond to fasting and time of the day, to understand whether any of these pathways are common to both exposures, converging downstream into a common pathway; 2) to identify biological processes shared by both skin and adipose tissue as this may hint at details of the systemic organization of the response to fasting and time of day; 3) to identify novel genes responding to fasting independently of time of day and genes robustly replicating over the literature in multiple tissues in humans, non-humans and across taxa (i.e. both humans and non-humans) as this may hint at functional and/or conserved pathways; 4) to assess the characteristics of common genetic variants regulating the expression of fasting responsive genes, by looking at expression quantitative trait loci (eQTL) and gene by environment (GxE) eQTL analyses.

## Results

**Fig. 1:**
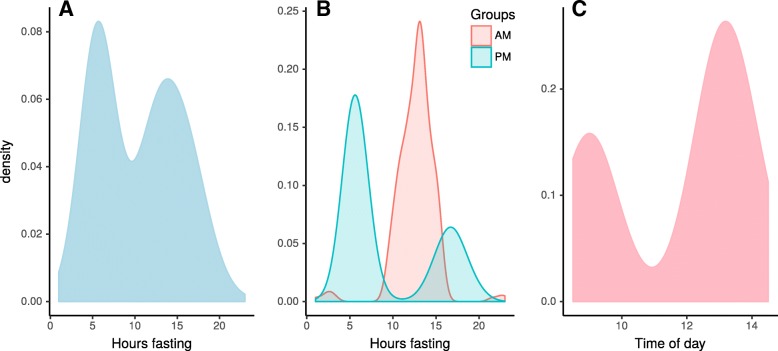
Overall distribution of **a** hours fasting, **b** hours fasting stratified by AM/PM, and **c** biopsy time of day in the 625 individuals participating in this study

We analysed 625 female twins cross-sectionally embedded in the TwinsUK registry that satisfied the inclusion criteria (the exclusion was mostly driven by having all variables collected at the same time point). Table 1 shows the characteristics of the individuals in this study. All individuals analysed are females with a mean age of 58 years and standard deviation (S.D.) of 9 years. The mean BMI was 27 kg.m^− 2^ and S.D. 5 kg.m^− 2^. This is in agreement with the rest of the UK population [24] for the age bracket. The hours fasted ranged from 1 to 23 h and the time of day when visits to the clinical facilities for the collection of the biopsy ranged from 08.30–14.30 h. The visits occurred relatively uniformly between Monday to Friday in ~ 226 days out of 365 calendar days for nearly 20 months. A total of 250 women attended clinic between 8:30 and noon (AM) and 444 attended between noon and 14:30 (PM). Individuals attending clinic AM were asked to fast overnight and PM individuals were asked to not eat after 9 AM. Self-reported hours fasting in AM, PM and overall varied considerably (Fig. 1) suggesting social desirability bias was small. Abdominal punch biopsies of subcutaneous adipose tissue (fat) and skin were extracted from a single punch biopsy taken during the clinical visit. RNA extraction, quantification and normalization were all performed as previously reported [25]. Expression levels were inverse-normal rank transformed to improve robustness of the analysis. We report our results at gene level for the best meta-exon, hereafter called exons unless otherwise stated. *P* values smaller than 10^− 16^ were rounded-up to *P* < 10^− 16^ avoid numerical precision errors.

**Table 1:** Study characteristics of the 694 females from the TwinsUK Cohort analyzed in this study

Variable	Mean	SD	IQR	N
Age	58.51	8.90	12.02	694
Hours fasting	10.42	4.74	9.00	694
Time of day	11.75	2.06	4.00	694
Glucose	4.91	0.42	0.60	656
Insulin	44.20	23.76	31.00	622
Total Cholesterol	5.57	1.03	1.40	678
HDL	1.81	0.43	0.61	675
Triglycerides	1.01	0.39	0.54	647
Height	161.43	6.04	8.70	681
Weight	69.37	14.12	16.40	681
BMI	26.59	5.08	5.88	681

### Transcriptional response to fasting is more extensive in adipose tissue

**Fig. 2:**
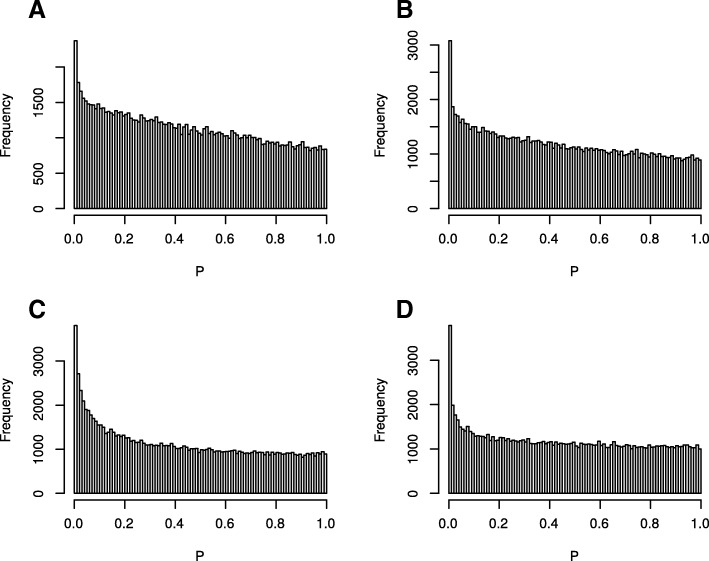
Histogram of the *P* values for the association between expression levels of all expressed genes in adipose and skin tissue with time of the day (TOD) and hours fasting (HF): **a** TOD in skin, **b** TOD in adipose, **c** HF in skin, **d** HF in adipose

To identify genes responding to hours fasting conditionally independent of time of the day, we regressed gene expression levels on hours fasting adjusting for time of the day, age, BMI, seasonality and other technical covariates using a mixed effects linear model (Fig. 2). All exons significantly associated (*P* < 10^− 5^ and FDR < 0.05) with hours fasting, time of day and/or season are reported in Additional file 1: Table S1. The response to hours fasting was more extensive and stronger in adipose than skin. We identified 367 genes responding to hours of fasting in adipose and 79 in skin (Table 2 and Additional file 1: Table S1). The magnitude of the fasting effect was larger in adipose than skin in the 29 genes significant in both tissues (Table 3, |β_fat_|-|β_skin_| = 0.017, mean magnitude difference t-test *P* < 10^− 16^**)**. The response to time of day affected substantially less genes than fasting in both adipose (99) and skin (54), despite both exposures being assessed simultaneously (Table 2). The overall mean magnitude of the response to time of day was similar across tissues (|B|⋍0.10, *P* = 0.7 and|β_fat_|-|β_skin_| = 0.001, *P* > 0.8) (Table 3). This suggests there are differences in which tissues respond to what systemic exposures and also differences in the magnitude of that response.

**Table 2:** Number of genes associated with hours of fasting or time of day in adipose and skin tissue

	Adipose		Skin		Both tissues
Exposure	Genes	Exons	Genes	Exons	Genes
Hours fasting	367	871	79	179	29
Time of day	99	211	54	127	17
Both exposures	71	150	19	73	15

Each row lists the number of genes and exons whose expression is associated to the listed exposure in adipose tissue, skin tissue or both adipose and skin tissue respectively. The final row lists the number of genes that are associated to both hours of fasting and time of day in adipose tissue, skin tissue or both tissues

**Table 3:** Magnitude of the effect size across tissues and exposures

	Mean|β|		S.D.		Cross - Tissue*
Exposure	adipose	Skin	adipose	Skin	P
Hours fasting	0.048	0.046	0.011	0.009	1.2E-03
Time of day	0.107	0.106	0.017	0.025	7.0E-01
Cross-Exposure P	7.1E-131	5.3E-58	N.A.	N.A	N.A.

^*^Statistical significance of the difference between tissues of the regression coefficient magnitude for the subset of exons significant in both tissues using an unpaired fisher t-test

**Fig. 3:**
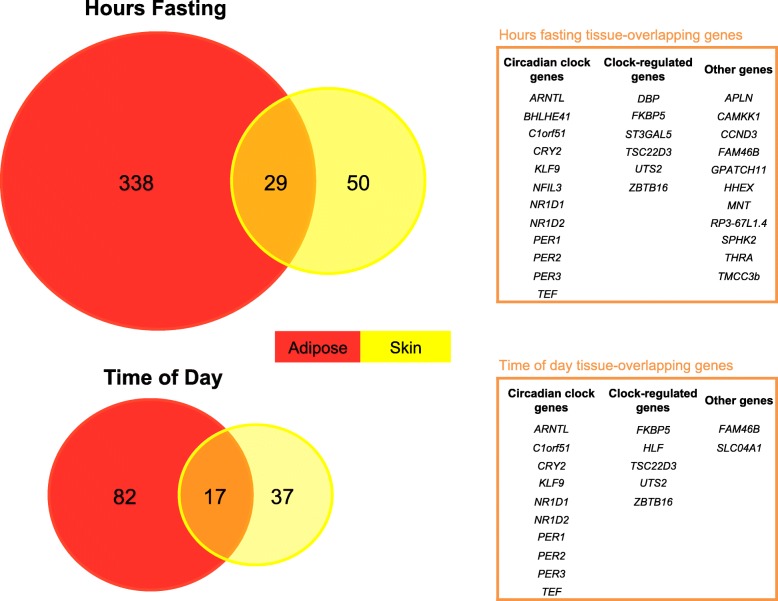
Tissue-specificity of gene expression response to hours of fasting and time of day. Venn diagram of genes associated to hours fasting (top) and time of day (bottom) in adipose tissue (red) and skin (yellow) tissue. Genes associated to an exposure in both tissues are listed in the tables on the right. Tissue-shared genes are annotated to indicate genes that are members of the circadian clock or known to be regulated by circadian clock genes

To identify tissue shared and tissue specific genes responding to hours of fasting and time of day, we looked directly at the overlap of significant associations by assessing the intersection of genes significant in both tissues (Table 2). We found 29 genes associated with hours fasting overlap across tissues (Overlap significance *P* < 10^− 16^, odds ratio (OR) =18.9) and 17 for time of day (Overlap significance *P* < 10^− 16^, OR = 60.3). This corresponded to 38 and 31% of the genes associated with fasting and time of day respectively (Table 2). The common genes in either tissue are related to circadian clock or involved in the regulation of the circadian rhythm (Fig. 3). The considerable overlap between tissues, particularly for hours fasting, suggest there is a systemic component in addition to the tissue-specific responses.

### Magnitude of the response to time of day is larger than hours fasting despite less genes associated

To identify the overlap of genes responding to both fasting and time of day, we intersected the number of genes associated with both exposures in each tissue. (Table 2). The significance of the overlap was inferred with a fisher test as above. We found 71 genes in adipose (Overlap significance *P* < 10^− 16^, OR = 37.1) and 19 in skin (Overlap significance *P* < 10^− 16^, OR = 67.4) that respond to both hours fasting and time of day. This corresponds to 72% of genes associated with time of day in adipose and 35% in skin (Table 2). The substantial overlap of genes that respond to hours fasting conditionally independent of time of day in adipose suggests that these exposures elicit similar downstream processes.

To assess and compare the magnitude of the response to hours of fasting and time of day, we compared the average magnitude of the effect size (expression standard deviations/hour) of the exons significantly associated with each exposure in adipose and skin (Table 3). We found that the magnitude of adipose and skin response to time of day was around twice that of hours fasting (|β_HF_| ≅ 0.05 and|β_TOD_| ≅ 0.1, difference in mean t-test *P* < 2 × 10^− 16^). Time of day effect is in the opposite direction of fasting for all exons significantly associated with both exposures (Additional file 1: Table S1). These results suggest that the effect of time of day in gene expression is strong in these tissues, and may counteract the effect of fasting around the time clinical examinations occurred.

### Genes associated with hours fasting replicate among human tissues and across taxa

To replicate our results, and identify genes that are associated with hours fasting consistently across human tissues and taxa, we conducted a literature search for gene expression papers of diet restriction (including fasting modalities) and queried the GenDR database for genetic manipulations in model organisms that cancel out or disrupt the life-extending effects of diet restriction **(**Additional file 1: Table S2**)**. We looked at 3 types of replication: in human studies only, non-human studies only, or in both human and non-human studies. The later form of replication we termed “across taxa”. We assessed the replicability of each study by looking at the percentage of their genes replicated in other studies and likewise, we assessed the replicability of each gene by looking at the percentage of studies that reported the same gene (see methods). Among studies sampled from the literature, the median percentage of genes replicable in at least another study was 28.5% in humans and 57% in nonhumans. In both cases, studies on fasting and calorie restrictions show similar pattern of gene overlapping (Additional file 1: Table S2). In our study **(**Additional file 1: Table S3), 194 genes in adipose and 45 in skin replicated at least in one other study (Corresponding to 53% in adipose and 57% of genes in skin). In adipose 33 genes replicated across taxa, 24 in humans only, 137 in non-humans only (Additional file 1: Table S3). In skin 8 genes replicated across taxa, 3 in humans only, 34 in non-humans only (Additional file 1: Table S3). Four of the 19 genes associated to fasting in both tissues robustly replicate across taxa e: *PER1, NFIL3, CAMKK1, SPHK2*. *PPARG,* a key gene in adipogenesis and a Type 2 Diabetes drug target was implicated by our study in adipose tissue response to fasting, replicating previous results in model organisms. Our study implicates in the response to fasting for the first time 173 genes in adipose and 34 in skin. Novel genes in adipose include ageing-related *FOXO3*, and in skin *FOXN3-AS1*, a highly regulated non-coding gene with unknown function. Our replicability assessment shows that many genes responding to fasting in our study were replicated in multiple human tissues and are conserved across taxa, despite the multiplicity of designs and the fact that both studies in calorie restriction and fasting were included.

### Genes associated with hours fasting are enriched for circadian rhythm

**Fig. 4:**
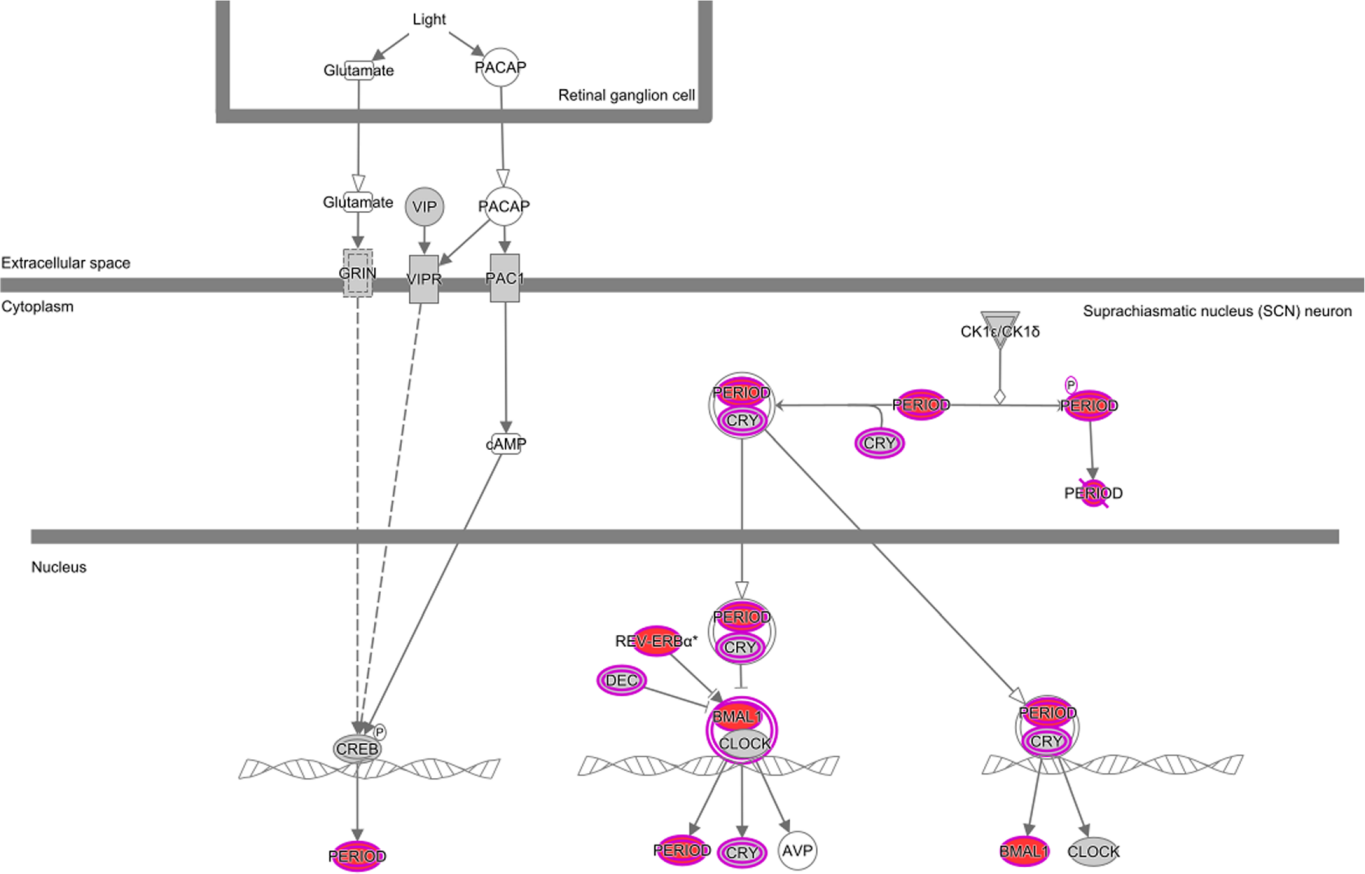
Expression levels of most genes involved in the circadian clock are associated with fasting in adipose tissue. Diagram shows the Ingenuity canonical pathway of the circadian clock annotated to highlight genes showing fasting-associated changes in fat gene expression in this study. Proteins (homo or heterodimer) with all coding genes associated to fasting are annotated in red, proteins with at least one gene associated with fasting are annotated with a pink border

We conducted a gene ontology enrichment analysis of the significant genes associated with hours fasting in adipose and skin using TopGO **(**Additional file 1: Table S4), ToppFun (Additional file 1: Table S5), and Ingenuity (Fig. 4, Additional file 1: Table S6). TopGO implements multiple correction algorithms for the hierarchical structure of the GO. The ToppFun database is not based on entrez and as such maps a larger number of genes than TopGO. Ingenuity database has proprietary pathways that are manually curated. We found that circadian rhythm and gene expression regulation of circadian rhythm were the top enriched ontologies in both adipose and skin for both time of the day and hours fasting (*P* < 2.3 × 10^− 05^, Fisher test BH FDR < 0.05). These results were robust to sensitivity analysis of the expression association *P*-value cutoff (Additional file 1: Tables S7, S8). The subset of genes associated with hours fasting in both skin and adipose (Fig. 3) yielded a stronger enrichment for circadian rhythm than each tissue separately (BH FDR < 0.05), suggesting a systemic response. The identified genes annotated with circadian rhythm gene ontology and ingenuity circadian clock pathway were assessed for replication in other studies. Eight genes of the circadian clock associated with fasting were replicated in other studies, 1 across taxa and the remaining 7 only in non-humans: *PER1*, *PER2*, *NR1D1*, *ARNTL*, *CRY2*, *BHLHE40*, *BHLHE41*, *NPAS2*. Among the genes responsive to the circadian rhythm, three replicated across taxa (*KLF10*, *CREM*, *NFIL3*) and three only in non-human studies (*DBP*, *KLF9*, *PPARG*). Novel circadian rhythm genes associated with fasting include *PER3*, *CIART*, *UTS* and *PTGER4*. The overall set of replicable genes were highly enriched for circadian rhythm GO term (BH FDR < 1.5 × 10^− 3^ in either adipose or skin).

In addition to the strong enrichment for circadian pathways, a range of other processes were also enriched in the genes associated to the two exposures (Additional file 1: Tables S4-S6). Focusing on the TopGO results, genes associated to fasting in adipose were enriched for glucose homeostasis (*P* = 1.6 × 10^− 08^), hormone secretion (*P* = 1 × 10^− 08^), regulation of vascular endothelial growth factor receptor signaling pathway (*P* = 6.4 × 10^− 07^). Genes associated to time of day were enriched for glucocorticoid signaling and response to lipid in both tissues, and to fat cell differentiation in skin only and a range of hormone and ketone processes in adipose only. Previous studies in skeletal muscle identified genes in the Hypoxia and Inflammatory Response pathways. In our study these GO Terms are not significantly enriched after multiple test correction. However, 5 genes in the hypoxia GO Terms (*BNIP3L*, *FAM162A*, *CBFA2T3*, *MYC*, *DDIT4*) and 4 in the inflammatory response GO Term (*PTGER4*, *C1QTNF3*, *ETS1*, *CRHBP*) were significantly associated with hours fasting in our study, interestingly the hypoxia genes were not associated with time of day (Additional file 1: Table S1).

### Circadian clock pathway response to fasting not mediated or confounded by cardio metabolic risk factors, AM/PM visit and gene sequence parameters

To assess whether the enrichment for circadian genes in fasting and time of day were mediated or confounded by cardio metabolic risk factors, AM/PM interaction, residual confounding, gene sequence characteristics, and quantification method, we re-ran several models adjusting for several potential confounders **(**Additional file 1: Table S9) and repeated the enrichment analysis using TopGO in adipose and skin (Additional file 1: Table S10). We found that circadian rhythm related pathways were always the top enriched GO term regardless of the model tested (Additional file 1: Table S10). Adjusting for glucose and total cholesterol (which together with BMI and age explains hours fasting association with triglycerides, insulin, TC, LDL-C and HDL-C levels, see methods and Additional file 1: Table S11) did not change the ranking of the top gene ontologies, which means enrichment is not mediated or confounded by these metabolic pathways. We stratified the data into AM and PM samples and the enrichment was still present in PM (AM sample was smaller). As restricting the sample to individuals that attended PM visits controls the effect of the time of the day, residual confounding with this covariate can be excluded as a potential explanation for the association of hours fasting with gene expression levels. We then adjusted Gene *P*-values for the number of exons, gene length, GC content, etc. (see methods) but this did not change the result. We concluded from this analysis that circadian rhythms in gene expression are influenced by hours fasting independently of cardio metabolic risk factors and not confounded with the technical factors and parameters that we can sensibly implicate in the expression response of these tissues.

### Relationship between fasting and genetic regulation of gene expression

To investigate whether change in expression of the genes responding to fasting and time of day may have a role in disease and other phenotypes, we investigated whether *cis*-eQTLs of the exposure-responsive genes are coincident with complex trait GWAS signals. As GWAS-coincident *cis*-eQTLs place modulation of expression of these genes on the molecular pathway between the genetic variant and trait, it is plausible that fasting or time of day-induced changes in expression could also impact the linked trait. Previous *cis*-eQTL scans of this dataset [25] identified 195 independent regulatory variants at 195 of the 553 exposure-responsive genes; we identified complex trait GWAS signals coincident with 11 of these 165 regulatory variants (or proxies R^2^ > 0.8) with the Phenoscanner portal [26] (Table 4, Additional file 1: Table S12). Five genes responsive to hours of fasting (*CTPS1, DNM3, HEMK1, SLA3R2, TRIM27, TSEN15*) are regulated by *cis*-eQTLs coincident with height GWAS signals (Table 4). Fasting responsive genes were also regulated by variants associated to Crohn’s disease (*PTGER4),* Rheumatoid arthritis (*PHLDB1*), Lung Function (*ADAM19*) and with cardiac troponin (*TNNT2*). *NFAIP8,* which is responsive to time of day in adipose tissue, is regulated by a Waist-hip ratio associated variant.

**Table 4:** Fasting or Time of day response genes with regulatory variants overlapping GWAS signals of complex traits

Tissue	Exposure	GeneName	eQTL SNP	GWAS SNP	r2	Trait
Fat	Hours fasting	*ADAM19*	rs11134789	rs2277027	0.99	Lung function FEV1/FVC Lung function FEV1/FVC in ever smokers
Fat	Hours fasting	*CTPS1*	rs12037263	rs2802551	0.98	Height
Fat	Hours fasting	*DNM3*	rs17277932	rs17277932	1.00	Height
Fat	Hours fasting	*HEMK1*	rs399484	rs399484	1.00	Height
Fat	Hours fasting	*PHLDB1*	rs10790255	rs45565037	0.86	Rheumatoid arthritis
Fat	Hours fasting	*PTGER4*	rs10440635	rs10440635	1.00	Crohns disease
				rs7720838	1.00	inflammatory bowel disease Irritable bowel syndrome Self reported allergy
Fat	Hours fasting	*TRIM27*	rs115864834	rs429369	1.00	Height
Fat	Hours fasting	*TSEN15*	rs1952256	rs1046934	1.00	Height
Fat	Hours fasting & Time of day	*SLC9A3R2*	rs3211995	rs3211995	1.00	Height
Fat	Time of day	*TNFAIP8*	rs11064	rs1045241	0.99	Waist hip ratio in females Waist to hip ratio adjusted for body mass index
Skin	Hours fasting	*TNNT2*	rs12564445	rs12564445	1.00	Cardiac Troponin T levels

**Fig. 5:**
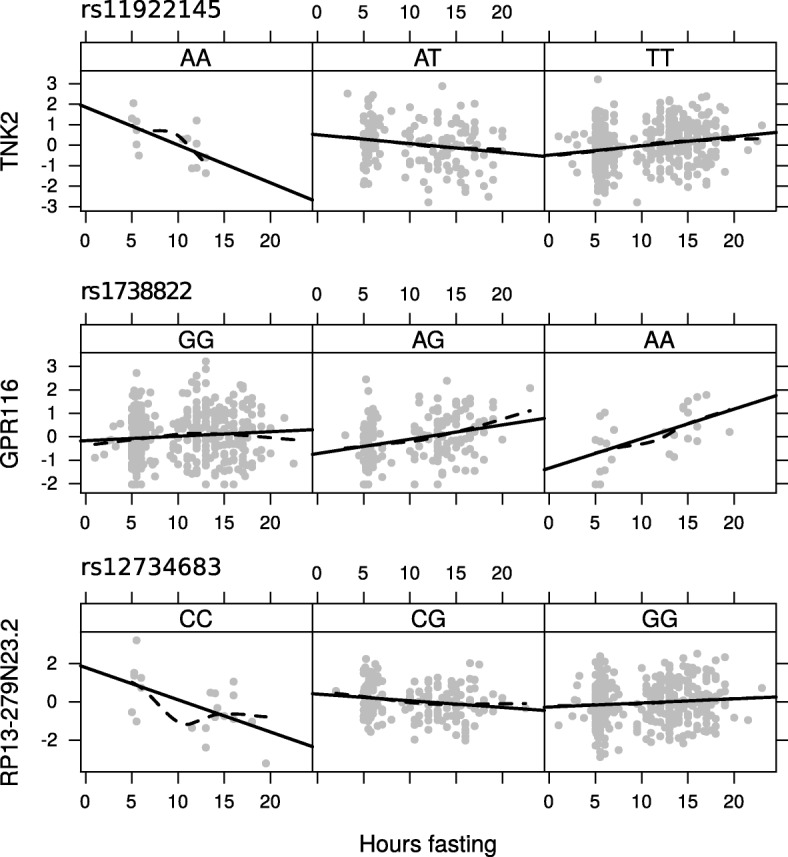
SNP x Hours of Fasting interactions on gene expression. The vertical axis represents expression of the given gene and the horizontal axis hours of fasting. Each point represents one individual. Each plot is split into three panels with individuals plotted in the panel corresponding to their genotype at the given SNP. Solid line represents the linear regression of expression on hours fasting, discontinuous line represents a smooth loess local regression

The response to external environments is not always uniform across individuals, and in some cases can be modified by the genotype of an individual. To identify common regulatory variants that modulate the response to hours of fasting or time of day we conducted a Gene x Hours-of-Fasting and Gene x Time-of-day *cis*-eQTL scan in each tissue. Overall, we identified 83 GxE interactions at an FWER < 0.05, with the largest number identified for Gene x Hours-of-Fasting in adipose (Additional file 1: Table S13). Looking at the 553 genes whose expression levels were associated to one of the exposures, we identified significant interactions at 15 genes (Table 5); 12 with hours fasting in adipose, one with time of day in adipose and one with time of day in skin (Three examples are plotted in Fig. 5). The significant overlap with exposure-responsive genes (OR = 7.6, *P* = 1.3 × 10^− 08^) suggest that genetic background may define the individual response to fasting and time of day at certain genes.

**Table 5:** Variants interacting with hours of fasting or time of day in fat and skin regulate expression of fasting or time of day associated genes

Tissue	Exposure	Gene	SNP	Chr	P	FWER
Fat	Fasting	*ACTA2*	rs813782	10	9.8E-06	1.0E-02
Fat	Fasting	*CBFA2T3*	rs12921348	16	5.6E-05	3.4E-02
Fat	Fasting	*FRMD4A*	rs10906711	10	2.9E-05	4.3E-02
Fat	Fasting	*HK2*	rs11690080	2	3.7E-05	3.0E-02
Fat	Fasting	*KIAA0182*	rs56377278	16	4.9E-05	4.7E-02
Fat	Fasting	*KLHL25*	rs11854452	15	1.9E-05	1.8E-02
Fat	Fasting	*MBD5*	rs13016227	2	3.0E-05	1.0E-02
Fat	Fasting	*PLXND1*	rs2403343	3	1.0E-04	2.9E-02
Fat	Fasting	*PPFIBP1*	rs405394	12	6.0E-05	4.8E-02
Fat	Fasting	*RP11-399O19*	rs813782	10	7.6E-05	4.4E-02
Fat	Fasting	*STARD13*	rs9591423	13	8.4E-05	4.7E-02
Fat	Fasting	*THRA*	rs11651987	17	2.4E-05	1.3E-02
Fat	TOD	*FZD10*	rs11061011	12	4.0E-05	3.4E-02
Skin	TOD	*HR*	rs10110714	8	7.6E-05	4.8E-02

## Discussion

We conducted an association study between gene expression and hours fasting, adjusting for time of day, seasonality, BMI, age and other technical covariates in a large sample of human adipose and skin biopsies. We identified 417 genes associated with hours of fasting in adipose and skin, which partly overlap with 137 genes associated to time of day in these samples. More than 50% of genes responding to fasting in our study were corroborated by a literature analysis of other gene expression studies on fasting and calorie restriction in other species and other human tissues. The response to hours fasting was more extensive in number of genes and stronger in magnitude in adipose tissue than skin, which is not unexpected given the central role of adipose tissue in metabolism. Interestingly, in genes associated to both exposures, the effect of time of day was stronger and in an opposite direction to the effect of hours of fasting, and thus may counteract the effect of fasting in some genes during the observed time window. This effect is consistent with clinical trials of the physiological response to meal times [27] suggesting improved cardio metabolic risk factors and weight management for those consuming most calories in the first half of the day. This finding may have consequences for deciding the optimal period of the day for fasting and planning of biomedical interventions.

Fasting responsive genes were enriched for circadian rhythm and circadian clock genes among other biological processes. The enrichment for circadian rhythm did not subside after sensitivity analysis, re-running the associations conditioning on cardio-metabolic risk factors, or when restricting to samples collected in the afternoon. Genes with transcriptional responses to both exposures in both tissues were even more highly enriched for circadian rhythms and circadian clock suggesting this is systemic component of the fasting response in adipose and skin of humans. A role of gene expression circadian rhythm in time-restricted diets has been hypothesized based on animal models and in vitro studies of human materials [2, 3] but the case for hours fasting is not yet fully established, particularly it was not clear whether the association is not confounded with time of the day, seasonality, and risk factors involved in the pathogenesis and pathophysiology of cardio metabolic disease. Our results contribute to this discussion by providing data from an observational study in multiple human tissues with approximately 625 individuals, the largest so far. Our results consolidate the theory that response to fasting and time of day share common pathways [4], particularly the circadian clock. The similarity of the beneficial effects on health of both time restricted diet and fasting might be underpinned by this common pathway, independently of known risk factors of the pathogenesis of cardio metabolic disease, such as cholesterol and triglycerides.

Regulation of genes response to time of day and hours fasting was tissue specific, with greater proportion of genes responding in fat, and interestingly the percentage overlap of genes responding to both exposures also larger in fat. We also found a larger proportion of regulatory variants interacting with hours fasting in fat; showing that expression plasticity in response to fasting is tissue specific. This support the notion that gene expression regulation is tissue-specific [28] and that tissue-specific regulatory variants may influence the onset of conditions affecting a particular tissue type. An idea supported by the fact that several exposure-responsive genes had regulatory variants overlapping GWAS signals of complex diseases.

Some limitations are important to mention, this is an observational twin-study of females with average age of 58 years and the hours fasting was self-reported which may be subject to error, recall bias, social desirability bias and self-selection bias. We removed individuals that fasted more than 23 h to avoid recall bias. Self-selection bias in hours of fasting, e.g. for medical reasons, is small as this is a relatively healthy sample. As the time of the visit was allocated more or less haphazardly, this exposure will be less affected by confounding. The study may have missed circadian rhythm genes with acrophase centred outside the collection interval or that respond less vigorously to time of day in these tissues. The circadian rhythm genes (particularly those in the canonical circadian clock) were the most significantly associated genes with time of the day (and with fasting duration), which indicate the response of at least these circadian rhythm genes was vigorous and centred in the sampling interval. In order to identify further circadian rhythm responsive genes, the biopsy collection needs to be extended over a broader time interval, including the night hours. In this case, additional genes whose expression oscillate in a sinusoidal pattern with a period of 24 h could be potentially captured using a cosinor transformation of time of the day. In our study, the collection time range is truncated to 6 h. Hence, mostly genes with moderate and large expression changes between morning and afternoon will be detectable. In this scenario, expending an additional degree of freedom in a cosinor model is less attractive due to the incurred loss of power to fit a more complex model and the risk of over-fitting a cross sectional sample. The fact that we detect most clock genes and large numbers of circadian rhythm genes suggests the linear model was a good compromise. As this is an observational study, individuals that fasted long hours tend to fast overnight. Consequently, there is a chance of a putative interaction or confounding between duration and modality of fasting, i.e. between fasting overnight and fasting long hours. Nevertheless, the effects can still be ascribed to fasting regardless of the underlying characteristic mediating the effect*.* While our sample size (*N* = ~ 625) is well powered to detect associations between gene expression and our exposures, it is relatively small for detection of genetic interaction effects, which limits the power of the GxE scan. GxE interactions are difficult to detect in humans, but recently have been detected on regulation of gene expression in several studies [29–31] where the reduced search space, large effect size and in some cases use of cell lines allows greater power to identify interactions. As such, we report the results of the GxE scan, but note these results will need to be tested in other datasets to determine their robustness. Some strengths of the study are also worth mention. As we carefully selected only external exposures and avoided endogenous or intermediate phenotypes, the study is more robust to collider bias and confounding.

## Conclusions

This study identified 417 genes whose expression is associated to hours fasting independently of time of day and seasonality, two important confounders of circadian rhythms. One hundred and seventy-eight of these genes have not previously been implicated in the response to fasting, and another 171 have not been linked to fasting in humans before. We identified tissue-shared and tissue-specific differences in the transcriptional response to fasting, and identified a core set of circadian rhythm genes that responding to both fasting and time of day in both adipose and skin tissue. This study contributes to the characterization of the transcriptional response to fasting in humans. Gene expression studies like this provide unique opportunities to unveil the molecular mechanisms of the response to exposures and therefore contributes to the identification of targets for therapy or drug development, and the conditions under which such targets vary.

## Methods

### Selection of individuals and embedded design

Twin pairs from the Twins UK registry were invited to clinical visit by telephone call and allocated to available time slots in the weekdays. The allocations happened haphazardly but subject to both twin pairs availability. Twins are routinely followed up over the years and multiple observations for some variables are available. We embedded a cross-sectional study on the subset of individuals whose materials for RNA extraction, hours fasting and clinical markers were collected on the same day that the anthropometric measurements were taken, with the exception of DNA which was collected previously. The rationale for using cross-sectional design follows directly from the time-dependent nature of the gene expression response to fasting and time of the day. We collected data over a period of 6 h which is 50% of the elapsed time between maximum and minimum amplitude (average 12 h for a period of 24 h). For the genes with acrophase at 8h30m or 14h30m the study can detect up to 50% of the total peak-to-peak difference (the maximum signal). We further restricted our analysis to individuals that fasted less than 23 h to avoid recall bias. Out of the ~ 850 individuals in all tissues, 625 individuals that meet all above criteria remained. Twins visits to the clinic were made over a period of ~ 600 days. The analyses of the collected materials were also performed at different times and in different batches. We recorded these dates and batches for investigation of potential confounders.

### Tissue collection

The study sample included 856 female twins who are a part of the TwinsUK registry and are all of European ancestry. Punch biopsies of subcutaneous adipose tissue from a sunlight-protected area of the stomach adjacent and inferior to the umbilicus were obtained from consenting individuals. Skin from the punch biopsy was then dissected to separate it from adipose tissue, and both samples were weighed and immediately frozen with liquid nitrogen. All the procedures followed were in accordance with the ethical standards of the St. Thomas Research Ethics Committee (reference 07/H0802/84) at St. Thomas Hospital in London. Volunteers gave informed consent and signed a consent form before the biopsy procedure. Volunteers were supplied with an appropriate detailed information sheet regarding the research project and biopsy procedure by post before attending the biopsy.

### Genotyping and imputation

Genome-wide SNP data for the TwinsUK individuals were generated as previously described. [32]In short, TwinsUK samples were genotyped on a combination of platforms (HumanHap300, HumanHap610Q, and 1 M-Duo Illumina arrays). Quality control and merging of the array datasets has previously been described in detail [32]. The cleaned data were pre-phased with IMPUTE2 with no reference panel and were then imputed into the 1000 Genomes phase 1 reference panel (interim, data freeze accessed November 10, 2010; the 1000 Genomes Project Consortium 2012). Variants with an INFO score > 0.8 on all platforms and a MAF > 5% were retained for analysis.

### RNA-Seq, exon quantification and normalization

RNA-seq data were generated, quantified and normalized as previously described [25]. In brief, samples were prepared for sequencing with the unstranded Illumina TruSeq sample preparation kit and sequenced on a HiSeq 2000 machine. The 49 bp paired-end reads were aligned to the UCSC Genome Browser GRCh37 reference genome with the Burrows-Wheeler Aligner. GENCODE v.10 was used to annotate genes. Samples were excluded if they failed to have more than 10 million reads map to known exons or if the sequence data did not correspond to actual genotype data. To quantify exons, all overlapping exons of a gene were merged into one meta-exon. We counted reads as mapping to a given meta-exon if either of its start or end coordinates overlapped a meta-exon boundary. All read-count quantifications were corrected for variation in sequencing depth between samples by normalizing the number of reads to the median number of well-mapped reads. We only used exons that were quantified in more than 90% of the individuals. Exon expression values were rank-based inverse normal transformed for downstream analysis.

### Covariates and clinical markers recorded

We considered the following previously collected anthropometric and metabolic markers: total cholesterol, total triglycerides, HDl cholesterol, glucose, and insulin. We also collect information on potential technical confounders, including the day of the year (1–365, for seasonality), age (years), day of the study (0–600 days), day the material was analysed in the lab (factor), day RNA-Seq was conducted (factor). The following RNA-Seq technical covariates were collected for each individual: GC content, median insert size, primer index.

### Transcriptome-wide association analysis of expression levels

Transcriptome-wide analysis were conducted using mixed effects models implemented in lmer package of R. The model used for all analysis unless otherwise stated is:

$$ {y}_i= XB+ Zu+{\in}_i $$

Where y_i_ is a vector of n individuals with the expression levels of exon i, X and Z are the design matrices of the fixed and random effects containing *n* individuals in the rows. The fixed effects include: hours fasting, time since last meal up to start of the clinical visit expressed in hours; *BMI*, the body mass index; *age*, years since born; time of day, local time of the clinical visit expressed in hours since midnight; Study day, number of days since the study started up to the day of the visit; the day of the year (ranging from 1 to 365, and cosinor transformed); percentage of GC content in the aligned reads of the RNA-Seq data; the median insert size. Hours fasting and time of clinical visit were modeled as continuous linear variables. The random effects were: Primer index; Date RNA-Seq experiment was conducted for this sample; Family identifier; Clonality identifier defined uniquely for each DNA sequence, such that a pair of MZ twins have the same id (because they have the same DNA) but each twin from a DZ pair have unique ids. These technical factors are an intrinsic part of the study design and should be included in the model. In addition, gene expression in this sample and others has been shown to be associated with age [33], BMI [31] and season [34]. As dawn time is a major cue for the synchronization of the circadian rhythm, we adjust for seasonality to avoid confounding with hours fasting and time of the day.

### Statistical significance of the gene expression association study

As expression levels are highly dependent, we corrected for multiple testing by applying the Benjamini Hochberg algorithm to hours fasting and time of the day (sample biopsy) in skin and fat and selecting the most stringent cut-off from all analyses (*P* < 1 × 10^− 5^) that guarantees a Benjamini Hochberg FDR ≤ 0.05 in all tissues and all exposures assessed.

### Replication of genes in the literature

We conducted two systematic searches in pubmed for papers reporting gene expression analysis of the response to fasting and calorie restriction separately in human and nonhuman mammals. As the automatic annotation of papers with MESH tags is not perfect, we enriched the search with the following strategies: i) retrieving all papers with “gene expression” keyword, selecting all relevant studies among the most recent top 100 papers; ii) using the same search string on google scholar, mendeley, and google. Once all relevant papers were identified, we extracted the significant genes reported in these studies, we converted probes, gene ids or gene symbols to ensembl ids using ensembl biomart. Studies reporting EST we used Genbank batch query followed by scripts developed in house to extract the RefSeq Id (e.g. NM_91388) and then converted these using the same process described above. Swissprot ids were converted to ensembl id using Swissprot tools. Non-human genes were mapped to ensembl ids using the strategies above and then mapped to human orthologues using ensembl biomart. Once all genes for all species were converted to human orthologues, we calculated the gene overlap between each study, including our study. We analysed two metrics to assess study replicability: Proportion of overlap between gene lists of study A and B was defined as|A∩B|/min(|A|,|B|); The percentage of the study genes replicated was defined as the number of genes that overlaps with at least another study divided by the total number of genes of study A_i_:|⋃_j_ (A_i_ ∩ A_j_)|/|A_i_|for all j ≠ i.

### Gene ontologies enrichment

We conducted gene ontology term enrichment using definitions of biological processes GO terms downloaded from R package org. Hs.eg.db, corresponding to a total number of 5828 in fat and 5992 in skin GO terms. All analyses restricted to terms < 500 genes. GO term enrichment was independently conducted on genes significantly associated with Hours fasting and Time of the day using the TopGO package from R. We implemented all enrichment tests present in the package. GO terms that pass bonferroni correction (*P* < 10^− 5^) either on Fisher Elimination or Fisher methods were considered significant but results for all algorithms are presented (mostly consistent across all methods). We repeated the enrichment analysis using Ingenuity and ToppFun due to quality of the annotation database. Except for ToppFun, all analyses were conducted considering only the genes expressed in the tissue. The gene annotations with the GO circadian rhythm and ingenuity circadian clock pathway were further confirmed against the literature.

### Sensitivity of GO term enrichment to *P*-value cut-off

We tested sensitivity of results to the significance cut-off by applying a more stringent cutoff on *P*-value and repeating the enrichment analysis. We considered two cutoffs: i) *P* < 2 × 10^− 6^, equivalent to at least Benjamini-Yekutieli FDR ≤ 0.05 in all tissues and all exposures and ii) *P* < 5 × 10^− 7^ corresponding to Bonferroni correction of at least 100,000 tests, which is approximately the total number of exons in fat and skin (114–116,000).

### Sensitivity to confounders of the power to detect expression associations

In order to test sensitivity to confounders of the power to detect associations with gene expression, we conducted a separate analysis correcting the *P*-value of the associations of exons with hours fasting by regressing the logit transformed *P* value on the percentage of GC ctranscriptontent in the DNA sequence of the gene, the gene length in bp, the number of isoforms reported for this gene, the number of meta-exons, the number of independent meta-exons (calculated with Ji and Li method using eigendecomposition of expression levels of all meta-exon in a gene) and adjusted for the following random intercept effects: Gene type, gene status and gene annotation database as defined by GenCode version 10. The residuals of the regression model were then back-transformed and used to ascertain the total number of significant genes and the respective gene ontologies enriched.

### Identification of potential cardiometabolic risk factors mediating or confounding the expression response to fasting

We regressed Glucose, TC, TG, HDL-C on hours fasting adjusting for age, age2, BMI, time of day, and the following random effects: Family, Clonality, Biopsy Date using a mixed effects model. We identified the subset of variables that are potential mediators or confounders of the effects of hours of fasting in the circulating levels of these cardiometabolic markers by i) identifying those markers associated with hours fasting, ii) discarding all markers whose association with hours fasting was abrogated after adjusting for a significant cardiometabolic marker.

### Correction for population structure and eQTL analysis

We correct population structure and twin relatedness using ProbABEL [35] two-step residual-outcome mixed models approach. First, we regress expression levels on technical covariates and kinship matrix using a mixed effects model.

$$ \mathrm{Expression}= XB+u+y,y\sim N\left(0,S\right),u\sim N\left(0,{\sigma}_aK\right) $$

The fixed effect *X* matrix have GC content and Insert size mode variables in the columns and individuals in the rows. The random effects *u* matrix have the primer index and date of RNAseq analysis as a proxy for batch effects in the columns. The kinship matrix *K* was estimated from imputed genotypes with INFO> 0.80 and MAF > 5% using GEMMA as previously described [31]. The residuals of the regression y is the outcome of this step. This regression is repeated for the expression of all genes or exons. The outcome of this step is a matrix of residuals with exons in the columns and individuals in the rows Y = {y_1,_y_2, …,_ y_m_}. In the second step we regress the expression residuals *y* on SNP and additional fixed effect covariates using the linear regression implemented in MatrixEQTL [36]. As described above, the additional fixed effects covariates are: hours fasting, BMI, age, time of day, study day, the day of the year. We analysed two GxE eQTL models, Y = SNP + E + SNP x E + C, where C are the remaining covariates besides the environmental variable E in the GxE analysis. In one model the corresponding environmental variable E was hours fasting and on the other model time of day**.** Notice that *y* are the expression residuals corrected for population structure using the imputed genotype data as well as corrected for technical covariates using both fixed and random effects.

### Statistical significance of GxE eQTL

We conducted a permutation test by fixing all covariates and randomizing the expression levels. The permutation was synchronized across all exons to preserve transcriptome-wide correlation structure. On each permutation, we save the smallest *p*-value from all SNPs associated with each exon. In order to correct for the correlation structure between exons from the same gene, we grouped the exon permutation *p*-values by gene to produce a more stringent gene-level correction of *p*-values. The permuted *p*-values were compared to the association *p*-value to obtain the FWER.

## Additional file

Additional file 1: **Table S1.** Genes significantly associated with hours of fasting, time of day or seasonality in fat or skin. **Table S2.** Replicability of literature studies and our study across taxa. **Table S3.** Replicability of genes associated with hours fasting and time of day. **Table S4.** Gene ontology enrichment of the hours fasting and solar time in fat and skin using TopGO. **Table S5.** Gene ontology enrichment of the hours fasting and solar time in fat and skin using ToppFun. **Table S6.** Ingenuity canonical pathways enrichment of the hours fasting and solar time in fat and skin using Ingenuity software. **Table S7.** Sensitivity analysis of GO term enrichment using Cut-off *P* < 2E-6 equivalent to BY FDR < 5%. **Table S8.** Sensitivity analysis of GO term enrichment using Cut-off *P* < 5E-7 equivalent to Bonferroni correction for 100, 000 independent tests. **Table S9.** Number of genes associated with hours fasting as a function of tissue, quantification, regression model. **Table S10.** Top pathway obtained with enrichment analysis of Fat and Skin gene expression as a function of quantification, regression model, and *P*-value corrections. **Table S11.** Epidemiological identification of mediators of the response to fasting. **Table S12.** Genes with eQTL SNPs overlapping GWAS Signals associated with multiple phenotypes, enriched for cardio-metabolic and immune related phenotypes. **Table S13.** Genes with GxE eQTL SNPs with FWER< 0.05. (XLSX 440 kb)

## Abbreviations

AM: Ante meridiem
BMI: Body mass index
eQTL: Expression quantitative trait loci
G x E: Gene by environment interaction
OR: Odds ratio
PM: Post meridiem
TOD: Time of day
